# Assessing the Risks and Benefits of Hypotensive Anesthesia and Piezosurgical Instruments in Bimaxillary Surgery

**DOI:** 10.7759/cureus.67394

**Published:** 2024-08-21

**Authors:** Koji Yamamura, Kaoru Murakami, Yosuke Hirata, Yuriko Toeda, Yasushi Kimura, Chikashi Minemura, Hidetaka Yokoe

**Affiliations:** 1 Oral and Maxillofacial Surgery, National Defense Medical College, Tokorozawa, JPN; 2 Dentistry, Self-Defense Forces Central Hospital, Setagaya, JPN; 3 Oral and Maxillofacial Surgery, Fukaya Red Cross Hospital, Fukaya, JPN

**Keywords:** landiolol, tachycardia, blood loss, piezosurgical instrument, hypotensive anesthesia, bimaxillary surgery

## Abstract

The challenge in bimaxillary surgery lies in significant intraoperative bleeding, prompting various strategies to minimize blood loss. Among the methods considered for controlling intraoperative bleeding, hypotensive anesthesia and the use of piezosurgical instruments (Osada, Tokyo, Japan) have been explored. However, hypotensive anesthesia may have adverse effects on cardiac function, and surgical time is likely to be prolonged when using piezosurgical instruments. This study retrospectively examined whether the combined use of hypotensive anesthesia and piezosurgical instruments reduces intraoperative bleeding and whether the combination impacts cardiac function. The combination of hypotensive anesthesia and piezosurgical instruments significantly reduced intraoperative bleeding. Furthermore, the combination was associated with a significantly higher incidence of tachycardia, particularly with the use of nicardipine. Additionally, the combination significantly lengthened the duration of the surgery and may have increased the burden on cardiac function. Landiolol was effectively employed to manage tachycardia. When selecting hypotensive anesthesia as a means to limit bleeding, proactive preparation and preventive small-dose administration of landiolol could be beneficial in managing the potential occurrence of tachycardia.

## Introduction

Orthognathic surgery is a well-established method for correcting dentofacial deformities, often necessitating bimaxillary surgery [1]. Bimaxillary surgery, which combines mandibular and maxillary osteotomies, allows for the alteration of the occlusal plane and the three-dimensional repositioning of the jaws. Many of the patients undergoing bimaxillary surgery are healthy young adults with minimal systemic complications [2]. However, in recent years, there has been an increasing incidence of elderly patients undergoing bimaxillary surgery, potentially making perioperative management more challenging [3].

Bimaxillary surgery can be associated with significant blood loss and the need for transfusion, emphasizing the growing importance of exploring alternative measures with minimal adverse effects to reduce intraoperative hemorrhage [2,4].

One approach to mitigating blood loss during bimaxillary surgery involves the use of hypotensive anesthesia [1]. Hypotensive anesthesia is a technique in which blood pressure is intentionally lowered during surgery to reduce bleeding and improve surgical field visibility [1,5]. While its potential for reducing intraoperative bleeding has been recognized, studies examining its efficacy specifically in bimaxillary surgery have produced conflicting findings [1]. Moreover, concerns regarding vital organ hypoperfusion during hypotensive anesthesia have been raised in other studies [1,5]. Notably, cardiovascular complications significantly contribute to postoperative morbidity and mortality following non-cardiac surgeries [6]. These complications extend hospital stays, escalating healthcare costs, and constitute a leading cause of perioperative mortality [7]. Elderly patients, in particular, exhibit a higher prevalence of coronary artery disease, amplifying the risk of cardiac events during the perioperative period [8].

Piezoelectric instruments utilize ultrasonic microvibrations of 60-200 μm/s at frequencies of 24-29 kHz to cut mineralized tissue while leaving soft tissue unharmed. These instruments provide an alternative strategy for bleeding management, albeit with potential challenges such as prolonged surgical duration [9]. Further investigation is warranted to understand their impact on increased bleeding and cardiac function in relation to the extended surgical time. Both hypotensive anesthesia and piezoinstruments are being explored as distinct approaches, and a comprehensive, sequential evaluation of both methods is unprecedented in the existing literature.

Therefore, the aim of this study was to investigate whether the combination of hypotensive anesthesia and piezosurgical instruments affects blood loss during bimaxillary surgery while ensuring intraoperative safety. Particular attention was paid to changes in circulatory function during surgery to assess whether the combination of hypotensive anesthesia and piezosurgical instruments imposes undue cardiac stress.

## Materials and methods

This study received approval from the Ethics Committee of the Defense Medical University Hospital, Japan Permit Number, 4859. Data were collected from the medical records of 30 patients who underwent bimaxillary surgery between 2015 and 2023. To be included in the study sample, patients had to meet the following criteria: they were classified as having American Society of Anesthesiologists (ASA) physical status I or II and had no significant systemic illnesses, such as severe cardiovascular, respiratory, renal, or hepatic diseases, which were excluded from the ASA categorization. Examples of excluded conditions include uncontrolled hypertension, severe asthma, chronic obstructive pulmonary disease, and advanced diabetes mellitus. Additionally, preoperative electrocardiogram (ECG) examinations confirmed the absence of waveform patterns suggestive of severe ischemic heart disease in all patients, who were aged between 18 and 80 years and included both males and females.

In all cases, continuous monitoring of patients' vital signs, including electrocardiography, pulse oximetry, temperature probe, and capnography, was conducted during surgery and the recovery period. A transducer connected to an indwelling arterial catheter was used to continuously monitor mean arterial pressure (MAP) in subjects during surgery. In hypotensive anesthesia, the target MAP was set between 50 and 65 mmHg.

The anesthesia induction technique was uniform across all cases, with an induction dose of propofol administered at 2 to 2.5 mg/kg. Fentanyl at 1 μg/kg was used to suppress intubation responses. Rocuronium at 0.6 mg/kg was administered as a muscle relaxant. All patients received cefmetazole 1 g or cefazolin 1 g immediately after induction, and the same antimicrobial agent in the same dose was administered every three hours.

The primary anesthesia maintenance agents included propofol, sevoflurane, and desflurane. Remifentanil at 0.2 to 0.35 μg/kg/minute was administered for analgesia maintenance. In the hypotensive anesthesia group, medications such as nicardipine, a calcium channel blocker, or alprostadil alfadex, a prostaglandin E1 preparation, were continuously infused to maintain the target MAP and bolus dose as needed.

All patients underwent a Le Fort I osteotomy and a bilateral sagittal split osteotomy (BSSO). Xylocaine 2% with 1:80,000 epinephrine was used for local infiltration anesthesia of the lower jaw and upper jaw. Firm fixation of bone segments was achieved using plates and screws, with no intermaxillary fixation applied postoperatively. Piezosurgical instruments were used in all cases in the hypotensive anesthesia group (Osada, Tokyo, Japan). Dexamethasone 6.6 mg or betamethasone 2 mg was administered before the conclusion of surgery in all cases. No transfusions were administered to any of the patients.

Intravenous fluid, urine output, and blood loss were measured. Intravenous fluid measurements were taken and recorded by the anesthesia team, while urine output and blood loss were measured by nurses and recorded by the anesthesia team. To measure intraoperative blood loss, a consistent method was applied to all cases. It involved subtracting the volume of saline used for irrigation during suctioning, both during washing and cutting, from the total volume in the suction canister. Additionally, the increase in weight of the gauze used (1 g = 1 ml) was added to determine the amount of blood loss. Furthermore, preoperative and postoperative day one blood tests were conducted, and using the Hct value, the estimated perioperative blood loss was calculated according to the equation proposed by Gross [10].

Statistical analysis was performed using GraphPad Prism 8 (MDF, Boston, USA). The two anesthesia groups were compared using a one-way analysis of variance (ANOVA), with unpaired t-tests used as post hoc tests for age and BMI. Fisher's exact test was employed for the analysis of sex, while the Mann-Whitney U test was used for other variables. Fisher's exact test was also utilized for the analysis of the occurrence of intraoperative tachycardia and the use of hypotensive agents. All values are presented as the mean ± standard deviation (± SD). Statistical significance was defined as p < 0.05.

## Results

**Figure 1:**
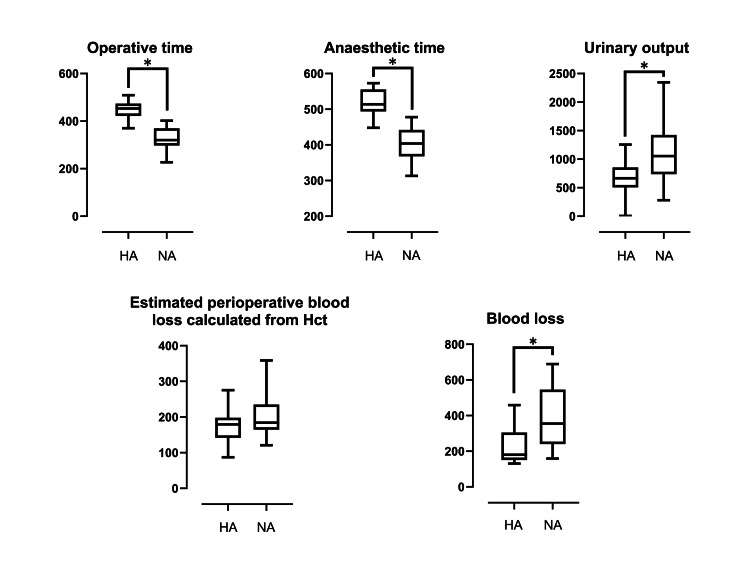
Comparisons of the outcome variables. HA: Hypotensive anesthesia, NA: Normal anesthesia, *p < 0.05 (Mann-Whitney U test).

An overview of the data is presented in Tables 1, 2, and Figure 1. There were no significant differences between the two groups in terms of age, BMI, or fluid replacement. The operative and anesthetic times were significantly longer in the hypotensive anesthesia group compared to the normal anesthesia group. Additionally, the urinary output in the hypotensive anesthesia group was significantly lower than in the normal anesthesia group. Concerning blood loss, the hypotensive anesthesia group exhibited significantly less bleeding compared to the normal anesthesia group. The estimated perioperative blood loss calculated from Hct values in preoperative and postoperative day one blood tests did not show a significant difference between the two groups.

**Table 1:** Comparisons of the outcome variables. BMI: Body mass index, Hct: Hematocrit

	Hypotensive anesthesia group (n = 13)	Normal anesthesia group (n = 17)	p-value
Variables	Mean ± SD	Mean ± SD	
Age (year)	27.5 ± 6.8	23.8 ± 5.6	0.1062
BMI (kg/m^2^)	22.4 ± 1.8	20.0 ± 4.7	0.0817
Fluid replacement (ml)	2635.7 ± 709.7	2462.5 ± 575.5	0.3291
Urinary output (ml)	684.1 ± 325.2	1170.6 ± 550.7	0.0045
Operative time (minutes)	447.2 ± 39.6	303.2 ± 90.8	< 0.0001
Anesthetic time (minutes)	521.2 ± 40.1	375.9 ± 107.3	< 0.0001
Blood loss (ml)	227.8 ± 112.2	387.8 ± 171.6	0.0059
Estimated perioperative blood loss calculated from Hct (ml)	174.6 ± 46.2	208.0 ± 69.9	0.3291

**Table 2:** Details on the use of landiolol for tachycardia. Landiolol maintenance dose (Mean ± SD): 6.75 ± 2.4 μg/kg/min MAP: Mean arterial pressure

Variables	Time after landiolol start (min)	
	0	10
	Mean ± SD	Mean ± SD
MAP (mm Hg)	61.3 ± 4.8	60 ± 1.6
Heart rate (bpm)	114.5 ± 6.6	94.3 ± 5.7

The hypotensive anesthesia group had a significantly higher incidence of tachycardia compared to the normal anesthesia group. Among the six cases where nicardipine was used, tachycardia occurred in three cases, suggesting a tendency for tachycardia to occur when nicardipine was used. No cases of tachycardia occurred when alprostadil alfadex was used. In the four cases where tachycardia occurred, adequate analgesia was confirmed before the administration of landiolol, and heart rate control was excellent in all cases.

As for postoperative complications, only one case of postoperative infection was observed in the hypotensive anesthesia group, and no other significant complications, except for nerve damage, were noted in either group.

## Discussion

Bimaxillary surgery involves various innovations to minimize blood loss, including the use of induced hypotensive anesthesia, ultrasonic cutting instruments, tranexamic acid, and local anesthesia [2,4].

In bimaxillary surgery, induced hypotensive anesthesia is administered using inhaled anesthetics, intravenous drug therapy, or a combination of both, taking advantage of the blood pressure-lowering effects of agents such as propofol and sevofluran [11,12]. Maintaining the desired hypotension during bimaxillary surgery can often be challenging, and in such cases, the concurrent use of drugs such as nicardipine, nitroprusside, labetalol, nitroglycerin, diltiazem, and prostaglandin E1 derivatives may be necessary [1,13,14]. Among these drugs, nicardipine is commonly used due to its affordability, accessibility, and ease of use [13]. Additionally, the use of piezosurgical instruments in bimaxillary surgery minimizes soft tissue damage and intraoperative blood loss but may extend surgical time, which is a drawback [4,15]. The extension of surgical time in bimaxillary surgery is known to significantly increase relative intraoperative blood loss [15]. In this study, the combination of hypotensive anesthesia and piezosurgical instruments resulted in significantly longer surgical times, likely attributed to the use of piezosurgical instruments, but did not result in increased blood loss. From these findings, it is suggested that the extension of surgical time with piezosurgical instruments may not be associated with an increase in intraoperative blood loss. This study used propofol and sevoflurane as maintenance anesthetics, and in cases where the target hypotension was not achieved, alprostadil alfadex (a prostaglandin E1 derivative) was used. The MAP was maintained at 50-70 mmHg during hypotensive anesthesia, which was consistent with values reported in other studies [1,16].

In this study, the hypotensive anesthesia group had significantly less blood loss compared to the normal anesthesia group, suggesting that the combination of hypotensive anesthesia and piezosurgical instruments was effective in reducing blood loss. While visual estimation is the most widely used method for measuring blood loss during surgery, it is not always accurate, especially in cases of hidden bleeding [17]. In such cases, several formulas using blood test results are used to estimate blood loss [17,18]. In this study, preoperative and postoperative hematocrit values were used to estimate blood loss, with the result that there was no significant difference in the estimated blood loss between the normal and hypotensive anesthesia groups. This outcome may be attributed to the fact that both groups received equivalent volume infusions; however, the normal anesthesia group exhibited a higher urinary output, resulting in an elevated postoperative hematocrit value, consequently leading to a lower calculated blood loss.

Hypotensive anesthesia, particularly in non-cardiac surgery patients, has been associated with adverse effects when the MAP falls below 60-70 mm Hg, even for short durations [19]. Prolonged maintenance of the MAP below 65 mmHg during surgery is known to increase the risk of organ dysfunction, with longer durations associated with higher postoperative mortality rates and organ dysfunction incidence [20]. Furthermore, a threshold estimated to cause a gradual increase in the odds of myocardial injury is reported to be a MAP of 65 mmHg during surgery [21]. It is recommended to maintain the MAP above 65 mmHg during surgery, especially after induction [22]. To avoid increasing oxygen consumption and causing ischemia in the unprepared heart, it is crucial to maintain a balance between myocardial oxygen supply and demand, which is best achieved by avoiding hypotension, tachycardia, and hypertension [23]. Maintaining cardiac output is particularly important for non-cardiac surgery patients [24]. The use of nicardipine has been associated with a relatively high incidence of reflex tachycardia, occurring in 17.2% of cases over a short period of time [13]. In this study, nicardipine was continuously administered for a relatively long period, leading to increased myocardial oxygen consumption and potentially a higher risk of circulatory dysfunction.

Beta-blockers are the preferred agents for managing tachycardia-induced arrhythmias, but their use can increase side effects such as stroke and severe bradycardia in low-risk patients, potentially worsening patient outcomes [25]. Among beta-blockers, esmolol and landiolol are short-acting, beta-1 selective agents, have no bronchoconstrictive effects, have very short half-lives, and provide rapid onset, allowing for continuous intravenous infusion and excellent adjustability [26]. Esmolol may be risky in cases with unstable hemodynamics due to its blood pressure-lowering effect, while landiolol effectively lowers heart rate without lowering blood pressure, making it favorable for rate control [27]. The landiolol group had a significantly higher rate of returning to sinus rhythm compared to the diltiazem group, with a lower incidence of hypotension and bradycardia, indicating safety [28]. Preventive landiolol administration has been suggested to be effective in preventing tachycardia in non-cardiac surgery cases [29,30]. However, there have been no reports of landiolol administration for intraoperative tachycardia in orthognathic surgery. In this study, landiolol was administered for the management of tachycardia, resulting in early improvement in circulatory function. Therefore, the administration of landiolol may be effective for patients undergoing orthognathic surgery who present with tachycardia.

Limitations of this study include the inability to discern the individual impacts of hypotensive anesthesia and the use of piezosurgical instruments, as they were employed concurrently. Consequently, it remains unclear which factor primarily contributed to the observed reduction in blood loss. Additionally, this study, being retrospective and relatively small in scale with a sample size of 30 patients, is inherently constrained by its methodology. Variations in anesthesia methods and hypotensive techniques further introduce potential confounding variables, both known and unknown. Moreover, the absence of a universally accepted gold standard for accurately calculating blood loss and estimating total blood volume necessitated the adoption of the formula proposed by Gross, which, while widely utilized, does not fully address the challenge of precise measurement in this context.

## Conclusions

The combination of hypotensive anesthesia and piezosurgical instruments during bimaxillary surgery effectively reduced blood loss. However, tachycardia may occur during hypotensive anesthesia, especially when nicardipine is used, and may lead to a strain on cardiac function. The study demonstrated the effectiveness of landiolol in managing tachycardia. For patients undergoing orthognathic surgery who present with tachycardia, the preparation and preventive use of landiolol may be beneficial.
